# Analysis of the Association of Administration of various glucocorticoids with development of acute pancreatitis using US Food and Drug Administration adverse event reporting system (FAERS)

**DOI:** 10.1186/s40780-019-0134-6

**Published:** 2019-02-28

**Authors:** Daisuke Nango, Yukifumi Hirose, Makoto Goto, Hirotoshi Echizen

**Affiliations:** 1 Departments of Pharmacy, Shin-Yurigaoka General Hospital, 255 Furusawa-tsuko, Asao-ku, Kawasaki, Kanagawa 215-0026 Japan; 2 Kyoto Constella Technologies Co., Ltd., 4th Floor, Kyozome Kaikan, 481 Tourouyama-cho, Nakagyo-ku, Kyoto, 604-8225 Japan; 3 Department of Pharmacotherapy, Meiji Pharmaceutical University, 2-522-1 Noshio, Kiyose, Tokyo, 204-8588 Japan

**Keywords:** FAERS, Glucocorticoids, Acute pancreatitis, Reported odds ratio (ROR)

## Abstract

**Background:**

There have been debates about the association between the administration of glucocorticoids and the development of acute pancreatitis, since many anecdotal cases of this adverse event were affected either by concomitant diseases (such as systemic lupus erythematosus, SLE) that may develop acute pancreatitis without glucocorticoid treatment or by co-administered drugs with high risk for the event. The aim of the present study was to explore whether disproportionally elevated signals of developing acute pancreatitis may be detected in patients receiving glucocorticoids as compared those receiving other drugs.

**Methods:**

We retrieved spontaneously reported cases of acute pancreatitis and clinically related adverse events (target events) from the US Food and Drug Administration Adverse Event Reporting System (FAERS) using 18 preferred terms (PTs). Target drugs studied were cortisol, cortisone, prednisolone, methylprednisolone, triamcinolone, dexamethasone, and betamethasone. After cleaning the data, we calculated reporting odds ratios (RORs) and 95% confidence intervals (CIs) of acute pancreatitis in patients who received one of the glucocorticoids. RORs were calculated for each glucocorticoid using all reported cases irrespective of reporters’ judgement about the contribution of the target drugs to events [i.e., primary suspected medication (PS), secondary suspected medication (SS), concomitant medication (C) and interacting (I)] and using cases with higher certainty of contribution (PS and SS), separately. When the lower limit of 95% CI of a ROR signal exceeded 1.0, the signal was considered statistically significant.

**Results:**

The RORs (95% CIs) calculated using all reported cases (PS, SS, C, and I) for cortisol (1.68; 1.43–1.98), prednisolone (1.33; 1.19–1.47), methylprednisolone (1.77; 1.55–2.02) were significant, whereas those for other target drugs were insignificant. Using the cases in which target drugs were considered to contribute the events with higher certainty (PS or SS), RORs for prednisolone (1.31; 1.10–1.55), methylprednisolone (1.62; 1.30–2.01), and dexamethasone (1.27; 1.10–1.47) were considered significant, whereas those for others were insignificant. Regarding the performance of PTs for detecting signals (RORs) associated with acute pancreatitis from FAERS database, “pancreatitis acute” gave RORs with higher significance than others, whereas more specific PTs, “haemorrhagic necrotic pancreatitis”, “ischaemic pancreatitis”, “pancreatic necrosis” and “pancreatitis necrotising”, gave RORs with greater magnitude.

**Conclusion:**

The present study demonstrated that the overrepresentation of signals for acute pancreatitis may be detected for prednisolone, methylprednisolone, and some others in the FAERS database.

(372 words)

## Introduction

A large number of cases that developed acute pancreatitis during treatment with glucocorticoids have been reported [1–14]. However, the causal relationship between the two remains controversial, since diseases with an indication for glucocorticoid therapy either as anti-inflammatory agents or antiemetics may have increased risk of developing acute pancreatitis. For instance, patients with systemic lupus erythematosus (SLE) may develop acute pancreatitis as a complication of systemic vasculitis [15], and patients with malignant neoplasia may concomitantly receive glucocorticoids as antiemetics and antineoplastic agents that may have high risk for acute pancreatitis (such as L-asparaginase and fluorouracil antineoplastics) [16]. Recently, the Ministry of Labour Health and Welfare has issued manuals of various severe adverse drug reactions (ADRs) for health professionals. As for the risk factors of acute pancreatitis, the authors described that there are many negative opinions on the causal relationship between the administration of glucocorticoid and development of acute pancreatitis [17]. Since severe acute pancreatitis could be fatal [18, 19], a rechallenge test is rarely undertaken for suspected cases of glucocorticoid-induced acute pancreatitis.

Analysis of drug-induced adverse events that have been archived in spontaneously reporting adverse drug reaction databases may be useful for detecting signals of specific adverse drug reactions in excessive frequencies over other adverse reactions [20–22]. Among the spontaneous reporting systems of adverse drug reactions operated by regulatory authorities of different countries, the US Food and Drug Administration Adverse Event Reporting System (FAERS) [23] is one of the biggest databases currently accessible to the public. In the present study, we aimed to study whether excessive signals of developing acute pancreatitis and its associated clinical conditions (including necrotizing and haemorrhagic pancreatitis) may be detected in patients receiving commonly prescribed glucocorticoids.

## Methods

We retrieved relevant datasets from the FAERS database compiled from the first quarter of 1997 to the first quarter of 2017. According to the Medical Dictionary for Regulatory Activities (MedDRA) version 20.1 [24], we employed 18 preferred terms (PTs) for collecting relevant cases associated with “acute pancreatitis [Standardized MedDRA Queries (SMQ): 20000022]” and its closely related clinical conditions. The SMQ codes for the corresponding PTs are shown in Table 1. According to the FDA recommendations, we removed duplicated data and irrelevant data from the retrieved data. In FAERS data, drugs may be documented under non-proprietary (generic) names, brand names, or their abbreviations. As a result, a drug may be filed under different synonymous names. In addition, an identical pharmaceutical molecule may be filed under different chemical names depending on different pharmaceutical products (such as cortisol and hydrocortisone). Furthermore, a glucocorticoid molecule may be used either as the free base or various salt forms in the products. For instance, hydrocortisone is formulated as free base as well as sodium succinate or sodium phosphate salts in different products. The same also applies to other glucocorticoids (including dexamethasone, triamcinolone, prednisolone, betamethasone, and methylprednisolone). As a result, concomitantly administered drugs in different cases under different synonymous names had to be unified into one of the typical drug names by text-mining approach. In the present study, multi-ingredient medications containing glucocorticoids and other ingredients (such as a combination formula of betamethasone and *d-*chlorpheniramine maleate) were excluded from analysis.

**Table 1:** Preferred terms (PTs) related to acute pancreatitis (SMQ;20000022)

Code	PT
10033625	Pancreatic haemorrhage
10033635	Pancreatic pseudocyst
10033636	Pancreatic pseudocyst drainage
10033645	Pancreatitis
10033647	Pancreatitis acute
10033650	Pancreatitis haemorrhagic
10033654	Pancreatitis necrotising
10033657	Pancreatitis relapsing
10048984	Pancreatic abscess
10052400	Oedematous pancreatitis
10056277	Pancreatorenal syndrome
10056975	Pancreatic phlegmon
10056976	Hereditary pancreatitis
10058096	Pancreatic necrosis
10059029	Cullen’s sign
10066127	Ischaemic pancreatitis
10075426	Grey Turner’s sign
10076058	Haemorrhagic necrotic pancreatitis

For pharmacovigilance analysis, several methods have been developed to detect overrepresented signals of specific adverse drug reactions for an individual drug [20–22]. In the present study, we employed reporting odds ratio (ROR) [20, 22]. RORs of acute pancreatitis in patients receiving various glucocorticoids were calculated according to the equation of Van Puijenbroek EP et al. [22]. Specifically, ROR was calculated as $\frac{a/b}{c/d}$; where “a” is the number of patients developing a target event (acute pancreatitis) when they received a target drug (glucocorticoid), “b” is the number of patients developing non-target adverse events, “c” is the number of patients developing the target event when they received non-target drugs, and “d” is the number of patients developing non-target adverse events when they received non-target drugs. In addition, 95% confidence intervals (CI) for the respective RORs were calculated by the following equation: $\mathit{\exp}\left[\mathit{\ln}(ROR)\pm 1.96\sqrt{\frac{1}{a}+\frac{1}{b}+\frac{1}{c}+\frac{1}{d}}\right]$. When the lower limit of the 95% confidence interval (CI) for a ROR was greater than 1.0, the signal was considered statistically significant. We calculated *p*-values of RORs with Fisher’s exact test. A p-value of < 0.05 was considered significant.

FAERS data contain not only information about all concomitantly administered drugs in patients who were reported to develop adverse reactions but also the reporters’ judgement about the contribution of each of the co-administered drugs to the adverse reaction. The reporters’ judgement about the certainty of the contribution of each drug to the corresponding adverse event was graded into four levels [primary suspected medication (PS), secondary suspected medication (SS), concomitant medication (C) and interacting (I)]. In the present study, we calculated RORs using all data irrespective of the subjective judgement about certainty (overall data) as well as RORs using only those data with higher levels of certainty (classified as PS and SS).

We further analysed the data to identify the PT that most effectively detects cases of acute pancreatitis. For this purpose, we used a volcano plot. Specifically, we plotted the negative common logarithm (to the base 10) of statistical significance (*p* values) on the y-axis and the normal logarithm (to the base e) of ROR on the x-axis for various PTs. This plot allows quick visual identification of data points displaying large magnitude signals that are also statistically significant. Statistical analyses were performed with JMP® Pro ver. 13 (SAS Institute Inc. NC, USA).

## Results

We listed all preferred terms (PTs) that were employed in the present study for identifying patients who developed acute pancreatitis (SMQ) and its related clinical conditions in Table 1. We retrieved a total of 10,413,882 adverse events from the FAERS database. After data cleaning, 8,437,343 cases were subject to analysis. We identified 16,431, 3580, 84,411, 11,363, 50, 242, 23,496, and 3825 cases who developed any ADRs while receiving cortisol, cortisone, dexamethasone, triamcinolone, prednisolone, methylprednisolone, and betamethasone, respectively. These numbers corresponded to 0.19, 0.04, 1.00, 0.13, 0.60, 0.28, and 0.05%, respectively, of the total cases of developing any ADRs that were used for analysis in the present study (i.e., 8,437,343 cases). A total of 44,893 cases were identified as developing acute pancreatitis (SMQ) while receiving any drugs including glucocorticoids. As shown in Table 2, 146, 22, 387, 50, 353, 220, and 24 cases of patients were reported developing acute pancreatitis (SMQ) while receiving cortisol, cortisone, dexamethasone, triamcinolone, prednisolone, methylprednisolone, and betamethasone, respectively. As a result, the numbers of these cases corresponded to 0.32, 0.05, 0.86, 0.11, 0.79, 0.49, and 0.05%, of the total cases of developing of acute pancreatitis (SMQ) (i.e., 44,893 cases), respectively.

**Table 2:** The numbers of cases of patients who developed acute pancreatitis (SMQ;20000022) during treatment with glucocorticoids and RORs for the respective glucocorticoids

	Total	Case	RR (%)	ROR (95%CI)	*P*-value
ALL (PS + SS + C + I)					
Cortisol	16,431	146	0.89	1.68 (1.43–1.98)	< 0.001
Cortisone	3580	22	0.61	1.16 (0.76–1.75)	0.49
Prednisolone	50,242	353	0.70	1.33 (1.19–1.47)	< 0.001
Methylprednisolone	23,496	220	0.94	1.77 (1.55–2.02)	< 0.001
Triamcinolone	11,363	50	0.44	0.83 (0.63–1.09)	0.20
Dexamethasone	84,411	387	0.46	0.86 (0.78–0.95)	< 0.01
Betamethasone	3825	24	0.63	1.18 (0.79–1.76)	0.38
PS + SS					
Cortisol	2865	16	0.56	1.05 (0.65–1.71)	0.80
Cortisone	640	2	0.31	0.59 (0.16–2.14)	0.78
Prednisolone	19,134	133	0.70	1.31 (1.10–1.55)	< 0.01
Methylprednisolone	9564	82	0.86	1.62 (1.30–2.01)	< 0.001
Triamcinolone	4749	4	0.08	0.16 (0.06–0.41)	< 0.001
Dexamethasone	28,140	190	0.68	1.27 (1.10–1.47)	< 0.01
Betamethasone	1121	11	0.98	1.85 (1.03–3.33)	0.058

Case; the number of reported cases with patients who developed acute pancreatitis during treatment with each glucocorticoid (target drug), Total; number of reported cases for the corresponding glucocorticosteroids, *RR* reporting ratio (case/total×100), *ROR* reporting odds ratio, *CI* confidence interval, *PS* primarily suspected drug, *SS* secondarily suspected drug, *C* concomitant drug, *I* interacting drug

The results of analysis showed that RORs calculated for cortisol, prednisolone, and methylprednisolone were significantly (*p* <  0.01) elevated when using data including all levels of certainty (Table 2), whereas the signal of dexamethasone was significantly less than the unity. In contrast, when analysis was performed using only the data with higher certainty for the association of glucocorticoids with adverse events, RORs for prednisolone, methylprednisolone and dexamethasone were considered significantly elevated (*p* <  0.01). We considered that the signal for betamethasone was insignificant. The *p*-value of the ROR calculated with Fisher’ exact test was slightly greater than 0.05, although the lower limit of the 95% confidence interval was greater than 1.0 (Table 2). The signal for triamcinolone was significantly less than the unity. In addition, there was an inconsistency about the RORs of dexamethasone that were calculated with use of ALL data and PS + SS data.

In Table 3, we showed the results of disproportionality analyses performed with use of various PTs for the three glucocorticoids (i.e., prednisolone, methylprednisolone, and dexamethasone) which showed significantly overrepresented signals for acute pancreatitis (SMQ). The RORs calculated for the combinations of prednisolone or methylprednisolone and two PTs (pancreatitis acute and pancreatitis necrotizing) were significantly (*p* <  0.01) elevated irrespective of the reporters’ certainty about the causality (i.e., ALL and PS + SS) (Table 3). In contrast, the results of the analyses for other three PTs (pancreatic necrosis, ischaemic pancreatitis and haemorrhagic necrotic pancreatitis) were inconclusive due mainly to a scarcity of samples.

**Table 3:** The numbers of cases who were reported as developing various PTs while receiving each of the glucocorticoids and RORs for the respective combinations of PTs and drugs

Drug	Case		ROR (95%CI), *P* value			
	ALL	PS + SS	ALL		PS + SS	
Pancreatitis acute (PT; 10033647)						
Prednisolone	159	64	2.41 (2.06–2.82)	< 0.001	2.53 (1.98–3.23)	< 0.001
Methylprednisolone	103	52	3.33 (2.74–4.05)	< 0.001	4.13 (3.14–5.42)	< 0.001
Dexamethasone	133	76	1.19 (1.00–1.41)	0.051	2.04 (1.63–2.56)	< 0.001
Pancreatitis necrotizing (PT; 10033654)						
Prednisolone	13	6	2.02 (1.18–3.46)	0.017	2.44 (1.12–5.33)	0.040
Methylprednisolone	7	5	2.32 (1.12–4.80)	0.035	4.07 (1.73–9.54)	0.009
Dexamethasone	19	15	1.76 (1.12–2.76)	0.021	4.18 (2.52–6.91)	< 0.001
Pancreatic necrosis (PT; 10058096)						
Prednisolone	9	5	5.99 (3.12–11.51)	< 0.001	8.63 (3.66–20.36)	< 0.001
Methylprednisolone	8	5	11.37 (5.70–22.68)	< 0.001	17.29 (7.33–40.80)	< 0.001
Dexamethasone	2	1	0.77 (0.21–2.81)	1.000	1.15 (0.20–6.55)	0.58
Ischaemic pancreatitis (PT; 10066127)						
Prednisolone	0	0	NS	NS	NS	NS
Methylprednisolone	0	0	NS	NS	NS	NS
Dexamethasone	2	2	98.96 (17.47–560.60)	0.001	298.86 (52.75–1692.06)	< 0.001
Haemorrhagic necrotic pancreatitis (PT; 10076058)						
Prednisolone	4	4	60.71 (20.42–180.51)	< 0.001	160.02 (53.82–475.83)	< 0.001
Methylprednisolone	4	4	130.24 (43.80–387.27)	< 0.001	320.57 (107.80–953.33)	< 0.001
Dexamethasone	0	0	NS	NS	NS	NS

Case; numbers of cases reported as developing the respective PTs during the administration of each glucocorticoid (target drug), *ROR* Reporting Odds Ratio, *PS* Primary suspect drug, *SS* Secondary suspect drug, *C* Concomitant drug, *I* Interacting drug, All; PS + SS + SC + I. RORs were calculated using data to which reporters had higher certainty about causality (PS + SS) and those included all levels of reporters’ certainty (ALL), separately, *NS* not significant

**Fig. 1:**
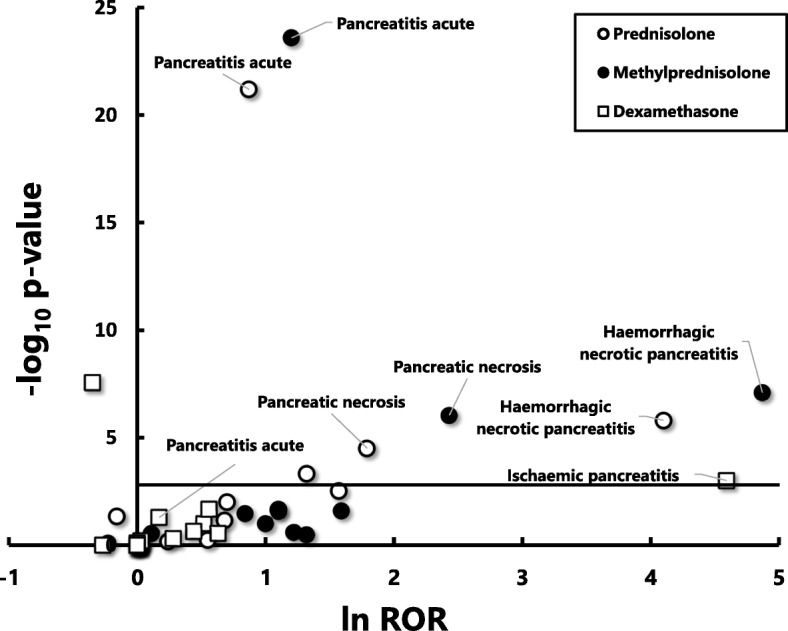
A volcano plot for visualizing statistical significance (*p*-values) and the magnitude of alarm signals (reporting odds ratios; RORs) for 18 PTs that were used for detecting the development of acute pancreatitis (SMQ) during the administration of prednisolone, methylprednisolone and dexamethasone. RORs were calculated using the reported data including all levels of reporters’ certain about the causality Negative common logarithm (to the base 10) of *p*-values (−log10 P) are plotted on the y-axis and natural logarithm (to the base e) of RORs (ln ROR) are plotted on x-axis. The horizontal line represents the threshold of significance (*p* = 0.05) corrected for multiple comparisons by Bonferroni’s method (*p* = 0.00093)

**Fig. 2:**
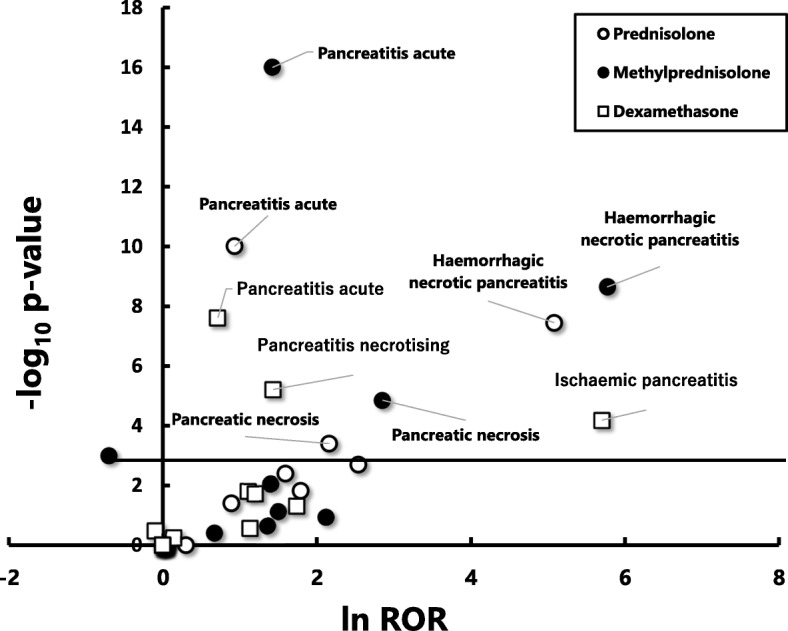
A volcano plot for visualizing statistical significance (*p*-values) and the magnitude of alarm signals (reporting odds ratios; RORs) for 18 PTs. RORs were calculated using the reported data to which reporters had higher certainly about causality (primary and secondary suspected) for prednisolone, methylprednisolone and dexamethasone Negative common logarithm (to the base 10) of p-values (−log10 P) are plotted on the y-axis and natural logarithm (to the base e) of RORs (ln ROR) are plotted on x-axis. The horizontal line represents the threshold of significance (*p* = 0.05) corrected for multiple comparisons by Bonferroni’s method (*p* = 0.00093)

We drew volcano plots for three glucocorticoids that showed statistically significant overrepresenting signals for acute pancreatitis (SMQ:20000022) when the analysis was conducted using data of all levels of reporters’ certainty about causality (Fig. 1) and when the analysis was conducted using data of higher certainty (Fig. 2). The plots showed that the PT of “pancreatitis acute” had the highest levels of statistical significance as compared other PTs, although the magnitude of the signal was less impressive than more specific PTs, irrespective of the reporters’ certainty about the causality between ADRs and administration of glucocorticoids (Figs. 1 and 2). In contrast, more specific PTs including haemorrhagic necrotic pancreatitis and ischaemic pancreatitis had greater changes in ROR signal, but their statistical significance was inferior to the PT of pancreatitis acute. Datasets for pancreatic pseudocyst drainage (PT; 10033636), pancreatorenal syndrome (PT; 10056277), pancreatic phlegmon (PT; 10056975), hereditary pancreatitis (PT; 10056976), Cullen’s sign (PT; 10059029), Grey Turner’s sign (PT; 10075426) were not plotted, since no cases were collected from the database.

## Discussion

To our knowledge, this is the first study to investigate the signals of developing acute pancreatitis during treatment with commonly used glucocorticoids, using a large spontaneous reporting system of adverse drug reactions, FAERS. We found significant overrepresentation of signals for acute pancreatitis (SMQ:20000022) over other adverse reactions for prednisolone, methylprednisolone. These findings were observed not only from the analysis using all data irrespective of reporters’ subjective judgment about the certainty for the contribution of glucocorticoids to acute pancreatitis, but also from the analysis using data judged by reporters as having higher levels of certainty (Table 2). In addition, we revealed that “pancreatitis acute” would be the best PT over others for detecting elevated signals associated with acute pancreatitis and related clinical conditions in the spontaneous reporting system, because volcano plots indicated that this PT showed by far higher significance based on *p*-value, albeit lower magnitude of changes in signal based on ROR, than more specific PTs including “haemorrhagic necrotic pancreatitis” and “ischaemic pancreatitis” (Figs. 1 and 2).

A diagnosis of drug-induced acute pancreatitis is often difficult to establish. Since acute pancreatitis is a rare and severe clinical condition with high mortality [18, 19], confirmation of a causal relationship between an assumed responsible drug and the event by rechallenge is difficult to conduct or ethically prohibited. In addition, patients who develop acute pancreatitis during treatment with a drug often have obvious risk factors for developing acute pancreatitis (such as alcoholism, systemic vasculitis due to immunological mechanism, and concomitant medications known to cause pancreatitis) other than the assumed perpetrator drug. Glucocorticoids have been claimed to be the aetiology of acute pancreatitis in patients receiving the drugs for the treatment of autoimmune diseases such as SLE. However, approximately 8% of patients with SLE develop acute pancreatitis irrespective of the administration of glucocorticoids [15]. Glucocorticoids were also implicated as the aetiology of acute pancreatitis in cancer patients who received glucocorticoids as antiemetic agent during anticancer chemotherapy. However, those patients are often given antineoplastic agents concomitantly, which are known to cause acute pancreatitis per se [16].

Besides anecdotal case reports of acute pancreatitis in patients receiving glucocorticoids, two lines of evidence may support the relationship between the administration of glucocorticoids and development of acute pancreatitis. Recently, we reported a patient with a diagnosis of autoimmune hepatitis (AIH) who developed acute pancreatitis immediately after administration of methylprednisolone [25] for the treatment of AIH. Since AIH has never been reported to be complicated with acute pancreatitis in the literature and the patient had no other possible causes of the event, we considered that there was a causal relationship between the administration of methylprednisolone and acute pancreatitis. Another approach to search for a signal of overrepresentation of acute pancreatitis during the administration of glucocorticoids over other drugs is using a large spontaneous reporting system of adverse drug reactions, such as FAERS. In the present study, we observed that RORs of acute pancreatitis for three glucocorticoids; prednisolone, methylprednisolone and dexamethasone, were significantly (*p* <  0.05) overrepresented with higher certainty (Table 2). Collectively, the present study may further support a causal relationship between the administration of glucocorticoids and the development of acute pancreatitis.

Badalov et al. [26] reported an evidence-based review on drug-induced acute pancreatitis. They reviewed literature data of drugs that were suspected to be associated with the aetiology of acute pancreatitis based on the weight of evidence for each agent and pattern in presentation. They classified drugs into four classes according to the certainty of the causal relationship. Class I drugs include medications in which at least one case report described a recurrence of acute pancreatitis with a rechallenge. No glucocorticoids were included in this class. Dexamethasone and prednisolone were classified into Class II, and other glucocorticoids were unclassified due to a lack of relevant data. Sadr-Azodi et al. [27] undertook a population-based nested case-control study in Swedish population and found that oral glucocorticoid use was associated with an increased risk of developing acute pancreatitis (odds ratio, 1.53; 95% CI, 1.27–1.84) compared with nonusers. Unfortunately, they did not analyse the contribution of individual glucocorticoids to the overall risk of acute pancreatitis separately. Collectively, these previous studies are in good agreement with the present study.

The present study provides an auxiliary finding about the selection of PTs for effective detection of a target adverse event. The volcano plots allowed visualization of the statistical significance and magnitude of signals for 18 PTs related to acute pancreatitis for prednisolone, methylprednisolone and dexamethasone simultaneously (Figs. 1, 2). The plots demonstrated that the PT, “pancreatitis acute”, detected signals (RORs) having by far the greatest statistical significance, albeit the less impressive magnitude in signal compared with others. In contrast, more specific PTs including haemorrhagic necrotic pancreatitis and ischaemic pancreatitis detected signals (RORs) with greater magnitude, but less significance, than the PT of “pancreatitis acute”. While this finding does not contradict our intuitive understanding, whether similar finding may be observed for the analysis of other drug-induced adverse reactions remains to be confirmed.

Spontaneous reporting systems for suspected adverse drug reactions are considered the cornerstone of pharmacovigilance, since they may detect potential alarm signals related to drug use. However, caution should be exercised when utilizing spontaneous reporting systems, since there are inherent limitations and obstacles (e.g., under-reporting, selective reporting, lack of information about total drug consumption, and many others) [28, 29]. As a result, the results of disproportionality analyses using spontaneously reporting ADR databases should be only considered as exploratory in a context of signal detection and it does not allow quantification of the true risk [28, 29] These limitations and obstacles may also exist in the present study. For example, clinical data of reported cases are often incomplete for detailed analysis in the present study. We were unable to search for clinical risk factors of acute pancreatitis including alcohol intake [30], the presence of cholelithiasis [31], hyperlipidaemia [32], obesity [33], and information of latency of the event. Also, information about concomitant medications such as immunosuppressive drugs (such as azathioprine [34]) that are classified as high risk (Class I) drugs of acute pancreatitis is often unavailable in the present study. Since these drugs may have been co-administered with glucocorticoids, further studies are required in the future.

In addition, there were some inconsistency among the results of the disproportionality analyses. As for dexamethasone, the ROR for acute pancreatitis (SMQ) that was calculated using the data including all levels of reporters’ certainly about the causality (i.e., ALL) was significantly less than the unity, but that calculated using the data for which reporters had higher certainty (i.e., PS + SS) was significantly greater than the unity (Table 2). We cannot offer any definitive explanations about this finding. Nevertheless, we recognized that the reporting ratio of acute pancreatitis (SMQ) for dexamethasone listed in Table 2 for the PS + SS data (0.68%) was approximately 50% greater than that for the ALL data (0.46%). This finding suggests that dexamethasone may have been reported more likely as primary suspected (PS) or secondary suspected (PS) drugs than as concomitant drug (C) or interacting drug (I) for concomitantly observed acute pancreatitis. Nevertheless, we cannot categorically negate a possibility that there may be a selective reporting bias of reporters due to their preconceived notion about a causality between the administration of steroids and development of pancreatitis.

## Conclusions

Using pharmacoepidemiologic approach, we demonstrated that there is increased risk of developing acute pancreatitis in patients receiving at least one of three glucocorticoids. While the present study does not prove the causal relationship between the administration of glucocorticoids and the development of acute pancreatitis, it may advance our understanding of this clinical issue.

## Abbreviations

CI: Confidence interval
FAERS: The Food and Drug Administration Adverse Event Reporting System
PT: Preferred Term
ROR: Reporting odds ratios
SLE: Systemic lupus erythematosus
SMQ: Standardized MedDRA Queries
SRS: Spontaneous reporting systems
